# Construction and application of a diagnostic model for tuberculosis in patients with pneumoconiosis

**DOI:** 10.3389/fmed.2026.1817334

**Published:** 2026-06-16

**Authors:** Shuang Qu, Shiping Hu, Jun Zhu, Huan Ye

**Affiliations:** 1 Department of Respiratory and Critical Care Medicine, Beijing Chest Hospital, Capital Medical University, Beijing, China; 2 Department of Respiratory and Critical Care Medicine, Fuxing Hospital, Capital Medical University, Beijing, China; 3 Department of Occupational Diseases, Shilong Hospital, National Center for Occupational Safety and Health, National Health Commission, Beijing, China

**Keywords:** machine learning, pneumoconiosis, risk diagnostic model, risk stratification, tuberculosis

## Abstract

**Objective:**

To develop and validate a machine learning-based model for diagnosing the presence of tuberculosis (TB) in patients with pneumoconiosis, utilizing complex clinical data to support early identification and clinical decision-making.

**Methods:**

This retrospective case-control study analyzed the risk of TB among patients with pneumoconiosis using data extracted from electronic medical records. A total of 325 patients with occupational pneumoconiosis who were admitted to Beijing Chest Hospital, Shilong Hospital, and Fuxing Hospital between January 2019 and June 2024 were included. Participants were classified into a case group (pneumoconiosis with TB) and a control group (pneumoconiosis without TB). Diagnostic variables included demographic characteristics, occupational exposure history, medical history, laboratory parameters, etiological indicators, and imaging features. The study outcome was the occurrence of TB. Multivariable regression analysis was performed to identify independent risk factors for concomitant pulmonary TB. Based on the selected variables, eight machine learning models were developed to construct diagnostic algorithms. Model performance was assessed using the area under the receiver operating characteristic curve (AUC), calibration curves, and decision curve analysis. The best-performing model was further interpreted using the Shapley Additive Explanations (SHAP) framework.

**Results:**

Regression analysis identified ten key diagnostic factors associated with TB in pneumoconiosis patients: etiological type, serum albumin, PaO_2_/FiO_2_ ratio, HbA1c, calcification, bilateral lung lesions, cavitation, prior antibiotic use, pleural effusion, and duration of dust exposure cessation. These diagnostic factors were further classified into three categories: occupational factors (etiological type, duration of dust exposure cessation), nutritional and metabolic indicators (serum albumin, PaO_2_/FiO_2_ ratio, HbA1c), and radiological features (calcification, bilateral lung lesions, cavitation, pleural effusion). Eight machine learning models were developed using these variables. After comprehensive evaluation of discrimination, calibration, clinical utility, and model stability, the random forest (RF) model demonstrated the best overall performance. The RF model achieved an AUC of 0.997 (95% CI: 0.994–1.000) in the training set and 0.905 (95% CI: 0.833–0.977) in the validation set, which outperformed other models.

**Conclusion:**

Patients with pneumoconiosis are at a high risk of developing TB. Machine learning models can effectively aid in identifying TB in this population, with the RF model showing superior diagnostic performance. This approach may facilitate early auxiliary diagnosis and timely clinical intervention.

## 1 Introduction

Occupational pneumoconiosis is one of the legally recognized occupational diseases in China and encompasses a group of chronic lung disorders characterized predominantly by progressive pulmonary fibrosis resulting from long-term inhalation of pathogenic mineral or organic dust during occupational exposure. According to current national occupational disease classification standards, pneumoconiosis includes multiple subtypes, such as silicosis, coal workers’ pneumoconiosis, and other dust-related interstitial lung diseases. With ongoing global industrialization, the incidence of pneumoconiosis has continued to rise worldwide (1–6). The sustained high disease burden has rendered pneumoconiosis a major public health concern, imposing substantial adverse effects on patients’ health, quality of life, and economic productivity, while also posing significant challenges to national healthcare systems and socioeconomic stability. Long-term dust exposure in pneumoconiosis patients causes persistent pulmonary inflammation, fibrosis, and structural lung damage, leading to impaired mucociliary clearance and weakened respiratory immune defense. Hence, these patients have markedly reduced resistance to infections, particularly *Mycobacterium tuberculosis*, which is the most common and severe complication of pneumoconiosis (7–9). Pneumoconiosis patients who develop tuberculosis (TB) often experience prolonged disease courses, higher recurrence rates, and poorer clinical outcomes, with some cases progressing to respiratory failure or death (10, 11). TB has thus become a major contributor to disease progression, increased mortality, and rising healthcare costs in this population. Effective prevention and timely management of *M. tuberculosis* infection are critical for reducing mortality, improving prognosis, and enhancing quality of life in patients with pneumoconiosis. This highlights the urgent need for reliable early diagnostic strategies for TB risk as a core component of comprehensive pulmonary infection prevention and control in this high-risk group.

Current evidence suggests that, in addition to occupational exposure, the risk factors for developing active pulmonary TB (PTB) in patients with pneumoconiosis largely overlap with those observed in the general population. These include smoking, alcohol consumption, malnutrition, diabetes mellitus, and human immunodeficiency virus (HIV) infection (9, 12). Among occupational and disease-related factors, the incidence of pneumoconiosis complicated by PTB has been shown to correlate with disease stage, age at onset, duration of dust exposure, and specific occupational exposure characteristics (13, 14). However, early diagnosis of active PTB in pneumoconiosis patients remains challenging. Clinical manifestations are often atypical, sputum bacteriological examinations may yield negative results, and radiological findings can be difficult to differentiate from pre-existing pneumoconiosis-related lung lesions. It is generally recognized that concurrent PTB should be suspected when pneumoconiosis patients present with acute symptom deterioration, rapid radiographic progression, or newly developed pulmonary nodules, consolidation, cavitary lesions, or pleural effusion on chest imaging (4, 15). However, most current risk assessment remains qualitative, highlighting the need for multivariate diagnostic models.

Over the past decade, artificial intelligence (AI) technologies have demonstrated substantial potential across multiple disciplines. Machine learning (ML), an interdisciplinary field integrating probability theory, statistics, optimization, and computer science, has emerged as a powerful approach for large-scale data analysis and pattern recognition (16). Common ML algorithms, including decision trees, support vector machines, and random forest (RF) classifiers, have shown strong diagnostic performance across diverse applications. ML-based approaches have exhibited significant advantages and promising clinical utility in pulmonary infectious diseases (17, 18). Zhang et al. (19) compared traditional logistic regression models with ML approaches and found that ML models can accurately diagnose treatment outcomes for patients with multidrug-resistant TB. Owing to their capacity to learn from complex, high-dimensional data, ML methods are expected to become important adjunctive tools in clinical diagnosis and management, particularly in early disease detection, personalized treatment strategies, and prognostic assessment. Furthermore, the Shapley Additive Explanations (SHAP) framework offers an intuitive way to interpret model decisions by quantifying each feature’s contribution to the diagnostic, thereby increasing the credibility and practical utility of ML models in clinical settings.

Despite growing recognition that pneumoconiosis complicated by TB results from the interplay of multiple intrinsic and extrinsic factors, effectively integrating these variables to achieve early risk diagnostic and timely diagnosis remains a major clinical challenge. China bears a high dual burden of pneumoconiosis and TB, and for this large and vulnerable population, rapid disease identification, early diagnosis, and standardized treatment are critical for improving clinical outcomes and quality of life. Therefore, bridging rapidly evolving diagnostic technologies with routine clinical practice to facilitate early screening and diagnosis of pneumoconiosis-associated TB is an important and urgent research direction. To date, no validated and explainable ML model exists specifically for patients with pneumoconiosis complicated by TB.

Based on this background, the present study was designed as a retrospective case-control investigation that systematically collected clinical, laboratory, and imaging indicators from patients with pneumoconiosis. By utilizing the strong learning capacity of machine learning algorithms and their ability to analyze complex clinical data, we aimed to construct a robust diagnostic model for TB risk in pneumoconiosis patients, thereby providing a scientifically sound and clinically useful tool to support early diagnosis and intervention in pneumoconiosis-associated TB.

## 2 Materials and methods

### 2.1 Study design and participants

This retrospective study collected clinical data from patients with occupational pneumoconiosis who were admitted to Beijing Chest Hospital, Shilong Hospital, and Fuxing Hospital between January 2019 and June 2024. All enrolled patients met the diagnostic criteria for occupational pneumoconiosis as defined by the Chinese National Occupational Health Standard GBZ70-2015 (*Diagnosis of Occupational Pneumoconiosis*). Patients in the TB group additionally fulfilled the diagnostic criteria for TB in accordance with the Health Industry Standards of China, including WS196-2017 (*Tuberculosis Classification*) and WS288-2017 (*Diagnosis of Pulmonary Tuberculosis*). Only patients with complete clinical records were eligible for inclusion. Patients were excluded if they had known immunodeficiency disorders, had received systemic glucocorticoids or immunosuppressive therapy within 6 months prior to admission, or had concurrent central nervous system infections or sepsis. Sample size estimation was performed using the widely accepted events-per-variable (EPV) principle, which recommends a minimum of 10 outcome events per independent variable included in diagnostic modeling. Given that this study anticipated incorporating approximately 10–15 independent variables, at least 150 cases of pneumoconiosis complicated by TB were required. A 1:1 ratio of cases to controls was applied, resulting in a minimum total sample size of 300 patients.

This study was approved by the Ethics Committee of Beijing Chest Hospital (approval number YJS-2025-06; July 16, 2025). All data were anonymized and analyzed in a digital format without disclosure of patient identifiers; therefore, the requirement for informed consent was waived.

### 2.2 Data collection and analysis

Clinical variables were collected at the time of hospital admission for all eligible pneumoconiosis patients. These included demographic characteristics such as sex, age, and body mass index; occupational exposure information including pneumoconiosis etiology, job type, duration of dust exposure, and disease stage; and preexisting clinical factors such as comorbid pulmonary diseases, smoking index, prior antibiotic use, and use of inhaled corticosteroids. Laboratory parameters comprised complete blood counts (including absolute white blood cell count, hemoglobin, platelets, neutrophils, and lymphocytes), liver function indicators (alanine aminotransferase, aspartate aminotransferase, and albumin), renal function indices (creatinine and blood urea nitrogen), arterial blood gas measurements (pH, PaO_2_, PaCO_2_, PaO_2_/FiO_2_ ratio, and lactate), glycated hemoglobin, C-reactive protein, procalcitonin, and erythrocyte sedimentation rate. Pathogen-related variables included the presence of co-infections. Imaging features assessed on chest examinations included lesion distribution, nodules, consolidation, cavitation, and patchy opacities. The primary study outcome was the diagnosis of TB during hospitalization, defined as an initial diagnosis made during the study admission.

All statistical analyses were performed using R software (version 4.4.1). Prior to analysis, the dataset underwent systematic preprocessing, including data cleaning, recording of categorical variables, and standardization and normalization of continuous variables to improve analytical performance. Variables were reviewed by clinical experts, and those lacking clinical relevance or diagnostic value were excluded. Missing data were assumed to be missing at random and were addressed using multiple imputation techniques.

Baseline demographic and clinical characteristics were compared between groups using the cleaned dataset. First, a normality test was performed on the data. Continuous variables with non-normal distributions were summarized as medians with interquartile ranges and compared using the Wilcoxon rank-sum test, while normally distributed variables were expressed as means with standard deviations and compared using independent-samples *t*-tests. Categorical variables were presented as frequencies and percentages and compared using the chi-square test. All statistical tests were two-sided, and a *P*< 0.05 was considered statistically significant.

### 2.3 Model development and validation

All eligible cases were randomly divided into training and validation datasets at a ratio of 7:3. Data from both the case and control groups were merged prior to splitting, resulting in a training set comprising 70% of the sample and an independent validation set comprising the remaining 30%. Baseline demographic and clinical characteristics were compared between the two datasets to ensure comparability and to assess the distribution of key variables.

Prior to model development, collinearity among candidate diagnostic factors was assessed using linear regression-based diagnostics, including evaluation of multicollinearity. Variables exhibiting significant collinearity were identified [variance inflation factor (VIF) > 5] and handled accordingly to minimize redundancy and instability in subsequent modeling.

All feature selection and model training procedures were performed exclusively using the training dataset. Candidate variables were screened using a combination of least absolute shrinkage and selection operator (LASSO) regression and univariable and multivariable logistic regression analyses. To reduce the potential randomness and instability associated with single LASSO feature selection, a nested cross-validation strategy was implemented to assess the robustness of variable selection. In the outer loop, the training dataset was randomly partitioned into five non-overlapping subsets for 5-fold cross-validation, with each subset sequentially used as a test set and the remaining subsets serving as training data. Within each outer training set, an inner 2-fold cross-validation loop was conducted to identify the optimal feature subset. Final diagnostic factors used for model construction were determined by integrating the results of LASSO regression and nested cross-validation.

Using the selected variables, multiple diagnostic models were constructed and compared. These included logistic regression, which estimates the probability of TB occurrence through a logistic function applied to a weighted linear combination of diagnostic factors; RF, an ensemble learning method that aggregates diagnostic from multiple decision trees; and support vector machine, which classifies samples by identifying an optimal hyperplane that maximizes the margin between classes. Additional models included k-nearest neighbors, which assigns class labels based on the majority vote among the closest neighbors in feature space; gradient boosting machine, which sequentially builds decision trees to correct residual errors; Light Gradient Boosting Machine (LightGBM), an optimized gradient boosting framework designed for efficiency and scalability; decision tree models, which apply hierarchical decision rules for classification; and Extreme Gradient Boosting (XGBoost), an advanced boosting algorithm that enhances diagnostic accuracy through regularization and optimized tree construction.

### 2.4 Model performance and clinical utility

Model performance was evaluated using the independent validation dataset. Discriminative ability was assessed using the concordance index, area under the receiver operating characteristic curve (AUC), sensitivity, specificity, and F1 score, with AUC serving as the primary evaluation metric. Model calibration and clinical utility were further examined using calibration curves and decision curve analysis (DCA). If model performance on the validation set was deemed unsatisfactory, resampling techniques were applied to address outcome imbalance, and the entire modeling workflow, including dataset splitting, feature selection, model training, and evaluation, was repeated to optimize diagnostic performance. To enhance model transparency and interpretability, the best-performing model was further analyzed using the SHAP framework.

## 3 Results

### 3.1 Description of baseline characteristics

A total of 325 patients with occupational pneumoconiosis were enrolled according to the predefined inclusion criteria. Among them, 218 patients with concomitant PTB were assigned to the case group, while 107 patients without TB comprised the control group. During data preprocessing, 19 variables with missing rates exceeding 40% were excluded from further analysis. These variables mainly included immune-related indicators, such as CD4^+^ cells, CD8^+^ cells, the CD4^+^/CD8^+^ ratio, B cells, natural killer cells, and inflammatory cytokines (IL-6, IL-8, and IL-10) (Table 1). For the remaining variables with incomplete data, missing values were imputed using a RF-based algorithm. Comparison between the original and imputed datasets demonstrated consistent distributional patterns, indicating that the imputation process did not introduce substantial bias (Tables 2,3).

**TABLE 1:** Data missingness of various variables.

Variable	Total number (*N* = 325)		Miss rate
	Significance	Missing number	
Age	325	0	0.00%
Sex	325	0	0.00%
BMI	325	0	0.00%
Etiology	325	0	0.00%
Type of work in production	325	0	0.00%
Exposure duration	325	0	0.00%
Pneumoconiosis staging	325	0	0.00%
Duration of dust exposure cessation	325	0	0.00%
Other underlying pulmonary diseases	325	0	0.00%
Smoking index	324	1	0.31%
Years of smoking cessation	324	1	0.31%
Antibiotic use	325	0	0.00%
Inhalation of hormones	325	0	0.00%
White blood cell	325	0	0.00%
Hemoglobin	325	0	0.00%
Blood cells	325	0	0.00%
NE#	325	0	0.00%
LY#	325	0	0.00%
MO#	325	0	0.00%
CRP	325	0	0.00%
PCT	263	62	19.08%
ESR	265	60	18.46%
ALT	325	0	0.00%
AST	325	0	0.00%
Albumin	325	0	0.00%
Creatinine	325	0	0.00%
Usea nitrogen	325	0	0.00%
CD4	12	313	96.31%
CD8	12	313	96.31%
CD4CD8	80	245	75.38%
B	62	263	80.92%
NK	61	264	81.23%
IL6	13	312	96.00%
IL8	10	315	96.92%
IL10	10	315	96.92%
TNFα	9	316	97.23%
PH	310	15	4.62%
PaO_2_	310	15	4.62%
PaCO_2_	310	15	4.62%
PaO_2_FiO_2_	310	15	4.62%
Lac	250	75	23.08%
Glycosylated hemoglobin	265	60	18.46%
Types of tuberculosis infection	325	0	0.00%
Tuberculosis drug resistance	325	0	0.00%
Tuberculosis treatment status	325	0	0.00%
NTM	325	0	0.00%
Germ	325	0	0.00%
Fungus	325	0	0.00%
Virus	325	0	0.00%
Location of CT lesions in the chest	325	0	0.00%
Tubercle	325	0	0.00%
Consolidation	325	0	0.00%
Cavity	325	0	0.00%
Patching	325	0	0.00%
Calcification	325	0	0.00%
Pleural effussion	325	0	0.00%
Aerothorax	325	0	0.00%
Bronchiectasia	325	0	0.00%
VC	21	304	93.54%
FEV1_A	81	244	75.08%
FEV1	78	247	76.00%
FVC	80	245	75.38%
FEV1FVC	82	243	74.77%
MMEF75/25	78	247	76.00%
DLCO	20	305	93.85%
Pulmonary arterial pressure	67	258	79.38%
End-diastolic diameter of right ventricle	65	260	80.00%
Main pulmonary artery diameter	149	176	54.15%

**TABLE 2:** Variable assignment table.

Dependent variable			
Column name	Variable name	Definition	Data status
Group	Is he infected with tuberculosis?	Classify	0 No 1 is
**Argument**			
**Column name**	**Variable name**	**Definition**	**Data status**
Age	Age	Quantify	−
Sex	Sex	Classify	1 Male 2 Women
Bmi	BMI	Quantify	−
Etype	Type of etiology	Classify	A. welders’ pneumoconiosis B. talc pneumoconiosis C. coal-worker’s pnuemoconiosis D. asbestosis E. cement pneumoconiosis F. blacklung
Exposure years (Eyears)	Exposure duration	Quantify	−
Stage	Stage of pneumoconiosis	Classify	A. 1 B. 2 C. 3
Free years (Fyears)	Duration of dust exposure cessation	Quantify	−
Other lungs (Olung)	Other underlying lung diseases	Classify	A. COPD B. Carcinoma of the lungs C. Pulmonary interstitial fibrosis D. Rheumatoid secondary pulmonary fibrosis E. Bronchiectasia F. Bronchial asthma G. other H. not have
Smoking	Smoking index	Quantify	−
Quit smoking (Qsmoking)	Years of abstinence	Quantify	−
Anti	Use of antibiotics	Classify	A. ≤ 3 Days/month B. ≥ 7 Days/month C. 3–7 Days/month D. Unused
Inhale	Inhalation of hormones	Classify	A. ≥ 14 Days/month B. Unused
WBC	White blood cell	Quantify	−
Hb	Hemoglobin	Quantify	−
PLT	Blood cells	Quantify	−
NE	NE#	Quantify	−
LY	LY#	Quantify	−
MO	MO#	Quantify	−
CRP	CRP	Quantify	−
PCT	PCT	Quantify	−
ESR	ESR	Quantify	−
ALT	ALT	Quantify	−
AST	AST	Quantify	−
Alb	Albumin	Quantify	−
Creatinine	Creatinine	Quantify	−
Urea	Usea nitrogen	Quantify	−
PH	PH	Quantify	−
PaO_2_	PaO_2_	Quantify	−
PaCO2	PaCO2	Quantify	−
PaO_2_ FiO_2_	PaO_2_/FiO_2_	Quantify	−
Lac	Lac	Quantify	−
HbA1c	Glycosylated hemoglobin	Quantify	−
Bacteria	Germ	Classify	A. Klebsiella pneumoniae B. SA C. Pseudomonas aeruginosa D. other bacteria E. not have
Fungus	Fungus	Classify	A. Monilia B. Aspergillus C. Other fungi D. Not have
Virus	Virus	Classify	A. Other viruses B. Not have
Location	Location of lesion	Classify	A. Bilateral lungs B. Right lung C. Left lung
Nodule	Tubercle	Classify	0 None 1 has
Consolidation	Consolidation	Classify	0 None 1 has
Cavity	Cavity	Classify	0 None 1 has
Patch	Patching	Classify	0 None 1 has
Cal	Calcification	Classify	0 None 1 has
Effusion	Pleural effussion	Classify	0 None 1 has
Pneumothorax	Aerothorax	Classify	0 None 1 has
Bronchiectasis	Bronchiectasia	Classify	0 None 1 has

**TABLE 3:** Analysis of baseline differences before and after interpolation.

	Fill before					After filling				
Variables	Total (*n* = 325)	Control group (*n* = 107)	Case group (*n* = 218)	Statistic	*P*	Total (*n* = 325)	Control group (*n* = 107)	Case group (*n* = 218)	Statistic	*P*
Age, mean ± SD	61.57 ± 10.99	66.01 ± 10.83	59.39 ± 10.41	*t* = 5.31	< 0.001					
BMI, mean ± SD	22.65 ± 3.54	24.34 ± 3.53	21.83 ± 3.24	*t* = 6.39	< 0.001					
Years of dust exposure, mean ± SD	16.22 ± 9.47	17.05 ± 8.85	15.82 ± 9.76	*t* = 1.10	0.274					
Years of dust exposure cessation, mean ± SD	16.51 ± 11.86	22.26 ± 12.07	13.68 ± 10.70	*t* = 6.25	< 0.001					
Smoking index, mean ± SD	295.34 ± 358.53	254.53 ± 322.80	315.46 ± 373.97	*t* = −1.44	0.151	295.65 ± 358.02	254.53 ± 322.80	315.83 ± 373.15	*t* = −1.453	0.147
Years of smoking cessation, mean ± SD	3.49 ± 7.70	6.14 ± 10.00	2.19 ± 5.87	*t* = 3.77	< 0.001	3.49 ± 7.69	6.14 ± 10.00	2.19 ± 5.86	*t* = 3.778	< 0.001
White blood cells, mean ± SD	6.79 ± 2.40	6.73 ± 1.88	6.82 ± 2.62	*t* = −0.36	0.717					
Hemoglobin, mean ± SD	134.29 ± 20.87	144.18 ± 15.79	129.43 ± 21.36	*t* = 7.01	< 0.001					
Platelets, mean ± SD	239.68 ± 86.74	218.63 ± 65.85	250.01 ± 93.75	*t* = −3.49	<0.001					
NE#, mean ± SD	6.81 ± 11.29	10.83 ± 18.78	4.84 ± 2.46	*t* = 3.29	0.001					
LY#, mean ± SD	2.16 ± 4.15	3.56 ± 6.35	1.48 ± 2.15	*t* = 3.31	0.001					
MO#, mean ± SD	0.75 ± 1.71	1.14 ± 2.76	0.56 ± 0.71	*t* = 2.14	0.035					
CRP, mean ± SD	22.87 ± 35.94	5.24 ± 11.00	31.53 ± 40.50	*t* = −8.94	< 0.001					
PCT, mean ± SD	0.12 ± 0.29	0.08 ± 0.23	0.13 ± 0.30	*t* = −0.99	0.321	0.11 ± 0.26	0.09 ± 0.15	0.13 ± 0.30	*t* = −1.128	0.260
ESR, mean ± SD	28.03 ± 28.84	15.66 ± 24.98	30.69 ± 28.97	*t* = −3.63	< 0.001	25.40 ± 26.69	14.61 ± 16.87	30.69 ± 28.97	*t* = −6.304	< 0.001
ALT, mean ± SD	21.53 ± 25.71	20.60 ± 16.34	21.99 ± 29.25	*t* = −0.46	0.646					
AST, mean ± SD	25.41 ± 21.76	22.03 ± 11.65	27.06 ± 25.15	*t* = −2.46	0.014					
Albumin, mean ± SD	37.95 ± 5.40	41.60 ± 3.98	36.16 ± 5.10	*t* = 10.52	< 0.001					
Creatinine, mean ± SD	68.64 ± 15.95	72.12 ± 13.69	66.94 ± 16.71	*t* = 2.78	0.006					
Urea nitrogen, mean ± SD	5.22 ± 1.88	5.80 ± 1.56	4.94 ± 1.96	*t* = 3.95	< 0.001					
PH, mean ± SD	7.41 ± 0.17	7.42 ± 0.02	7.41 ± 0.20	*t* = 0.37	0.713	7.41 ± 0.17	7.41 ± 0.02	7.41 ± 0.20	*t* = 0.363	0.717
PaO_2_, mean ± SD	83.30 ± 15.90	81.83 ± 12.91	83.92 ± 17.00	*t* = −1.06	0.292	83.24 ± 15.56	81.87 ± 12.07	83.92 ± 17.00	*t* = −1.250	0.212
PaCO_2_, mean ± SD	40.94 ± 5.00	40.09 ± 4.22	41.30 ± 5.26	*t* = −1.96	0.051	41.00 ± 4.89	40.40 ± 3.99	41.30 ± 5.26	*t* = −1.570	0.117
PaO_2_/FiO_2_, mean ± SD	358.03 ± 60.45	316.16 ± 69.63	375.70 ± 45.98	*t* = −7.54	< 0.001	358.36 ± 59.32	323.02 ± 67.43	375.71 ± 45.99	*t* = −7.292	< 0.001
Lac, mean ± SD	1.49 ± 1.46	1.23 ± 0.49	1.53 ± 1.55	*t* = −1.08	0.283	1.49 ± 1.29	1.41 ± 0.38	1.53 ± 1.55	*t* = −0.756	0.450
HbA1c, mean ± SD	5.94 ± 0.87	5.43 ± 0.67	6.05 ± 0.87	*t* = −4.59	< 0.001	5.86 ± 0.81	5.48 ± 0.46	6.05 ± 0.87	*t* = −7.742	< 0.001
Sex, n (%)				χ^2^ = 0.10	0.752					
Man	308 (94.77)	102 (95.33)	206 (94.50)							
Woman	17 (5.23)	5 (4.67)	12 (5.50)							
Type of disease, n (%)				−	< 0.001					
Welders’ pneumoconiosis	3 (0.92)	3 (2.80)	0 (0.00)							
Talc pneumoconiosis	1 (0.31)	0 (0.00)	1 (0.46)							
Coal-worker’s pnuemoconiosis	102 (31.38)	69 (64.49)	33 (15.14)							
Asbestosis	4 (1.23)	1 (0.93)	3 (1.38)							
Cement pneumoconiosis	2 (0.62)	0 (0.00)	2 (0.92)							
Silicosis	213 (65.54)	34 (31.78)	179 (82.11)							
Type of work in production, n (%)				−	<0.001*					
Electric welding	3 (0.92)	3 (2.80)	0 (0.00)							
mining/processing of metal ores	45 (13.85)	3 (2.80)	42 (19.27)							
Coal mining/processing	163 (50.15)	78 (72.90)	85 (38.99)							
Timber processing	5 (1.54)	1 (0.93)	4 (1.83)							
Gravel mining	58 (17.85)	8 (7.48)	50 (22.94)							
Stone processing	24 (7.38)	4 (3.74)	20 (9.17)							
Asbestos processing	4 (1.23)	1 (0.93)	3 (1.38)							
Feed culture	2 (0.62)	0 (0.00)	2 (0.92)							
Brickworks/Cement factories	21 (6.46)	9 (8.41)	12 (5.50)							
Pneumoconiosis stage, n (%)				χ^2^ = 23.65	< .001					
1	132 (40.62)	63 (58.88)	69 (31.65)							
2	83 (25.54)	23 (21.50)	60 (27.52)							
3	110 (33.85)	21 (19.63)	89 (40.83)							
Other underlying lung disease, n (%)				χ^2^ = 389.81	< 0.001					
Carcinoma of the lungs	5 (1.5)	1 (0.9)	4 (1.8)							
Pulmonary interstitial fibrosis	1 (0.3)	0	1 (0.5)							
Rheumatoid secondary fibrosis	1 (0.3)	0	1 (0.5)							
Other	19 (5.8)	0	19 (8.7)							
Not have	265 (81.5)	93 (86.9)	172 (78.9)							
Bronchiectasia	1 (0.3)	1 (0.9)	0							
Bronchial asthma	2 (0.6)	0	2 (0.9)							
COPD	31 (9.5)	12 (11.2)	19 (8.7)							
Use of antibiotics, n (%)				−	< 0.001					
Not used	288 (88.62)	80 (74.77)	208 (95.41)							
≤ 3 Days/month	10 (3.08)	2 (1.87)	8 (3.67)							
3–7 Days/month	25 (7.69)	25 (23.36)	0 (0.00)							
≥ 7 Days/month	2 (0.62)	0 (0.00)	2 (0.92)							
Inhaler use, n (%)				χ^2^ = 0.00	1.000					
≥ 14 Days/month	7 (2.15)	2 (1.87)	5 (2.29)							
Not used	318 (97.85)	105 (98.13)	213 (97.71)							
Germ, n (%)				−	< 0.001					
Klebsiella pneumoniae	29 (8.92)	19 (17.76)	10 (4.59)							
Staphylococcus aureus	2 (0.62)	1 (0.93)	1 (0.46)							
Other bacteria	85 (26.15)	15 (14.02)	70 (32.11)							
Pseudomonas aeruginosa	4 (1.23)	1 (0.93)	3 (1.38)							
Not have	205 (63.08)	71 (66.36)	134 (61.47)							
Fungus, n (%)				−	0.212					
Monilia	9 (2.77)	1 (0.93)	8 (3.67)							
Other fungi	9 (2.77)	2 (1.87)	7 (3.21)							
Aspergillus	5 (1.54)	0 (0.00)	5 (2.29)							
Not have	302 (92.92)	104 (97.20)	198 (90.83)							
Virus, n (%)				−	1.000					
Other viruses	2 (0.62)	0 (0.00)	2 (0.92)							
Not have	323 (99.38)	107 (100.00)	216 (99.08)							
Location of chest CT lesions, n (%)				χ^2^ = 80.45	< 0.001					
Bilateral lung	280 (86.15)	66 (61.68)	214 (98.17)							
Right lung	23 (7.08)	20 (18.69)	3 (1.38)							
Left lung	22 (6.77)	21 (19.63)	1 (0.46)							
Tubercle, n (%)				χ^2^ = 14.02	< 0.001					
Not have	55 (16.92)	30 (28.04)	25 (11.47)							
Have	270 (83.08)	77 (71.96)	193 (88.53)							
Consolidation, n (%)				χ^2^ = 3.87	0.049					
Not have	266 (81.85)	94 (87.85)	172 (78.90)							
Have	59 (18.15)	13 (12.15)	46 (21.10)							
Cavity, n (%)				χ^2^ = 42.02	< 0.001					
Not have	248 (76.31)	105 (98.13)	143 (65.60)							
Have	77 (23.69)	2 (1.87)	75 (34.40)							
Patching, n (%)				χ^2^ = 12.88	< 0.001					
Not have	128 (39.38)	57 (53.27)	71 (32.57)							
Have	197 (60.62)	50 (46.73)	147 (67.43)							
Calcification, n (%)				χ^2^ = 17.21	< 0.001					
Not have	217 (66.77)	88 (82.24)	129 (59.17)							
Have	108 (33.23)	19 (17.76)	89 (40.83)							
Pleural effusion, n (%)				χ^2^ = 34.85	< 0.001					
Not have	236 (72.62)	100 (93.46)	136 (62.39)							
Have	89 (27.38)	7 (6.54)	82 (37.61)							
Aerothorax, n (%)				χ^2^ = 0.21	0.646					
Not have	319 (98.15)	104 (97.20)	215 (98.62)							
Have	6 (1.85)	3 (2.80)	3 (1.38)							
Bronchiectasis, n (%)				χ^2^ = 0.00	1.000					
Not have	312 (96.00)	103 (96.26)	209 (95.87)							
Have	13 (4.00)	4 (3.74)	9 (4.13)							

t, *t*-test, χ^2^, Chi-square test, −, Fisher exact, *, Simulated *p*-value. SD, standard deviation.

Baseline comparisons revealed statistically significant differences between control groups and the case in 31 variables (*P* < 0.05). Notable differences were observed in age (66.01 ± 10.83 years vs. 59.39 ± 10.41 years, *P* < 0.001), body mass index (24.34 ± 3.53 vs. 21.83 ± 3.24, *P* < 0.001), duration of dust exposure cessation (22.26 ± 12.07 years vs. 13.68 ± 10.70 years, *P* < 0.001), hemoglobin concentration (144.18 ± 15.79 g/L vs. 129.43 ± 21.36 g/L, *P* < 0.001), and C-reactive protein levels (5.24 ± 11.00 mg/L vs. 31.53 ± 40.50 mg/L, *P* < 0.001). In contrast, 12 variables showed no statistically significant differences between groups, including years of dust exposure (17.05 ± 8.85 years vs. 15.82 ± 9.76 years, *P* = 0.274), smoking index (254.53 ± 322.80 vs. 315.46 ± 373.97, *P* = 0.151), and white blood cell count (6.73 ± 1.88 × 10^9^/L vs. 6.82 ± 2.62 × 10^9^/L, *P* = 0.717).

After data imputation, the overall statistical trends of most variables remained consistent with the pre-imputation analysis. Variables such as smoking index (254.53 ± 322.80 vs. 315.83 ± 373.15, *P* = 0.147), procalcitonin (0.09 ± 0.15 ng/mL vs. 0.13 ± 0.30 ng/mL, *P* = 0.260), and arterial pH (7.41 ± 0.02 vs. 7.41 ± 0.20, *P* = 0.717) continued to show no significant intergroup differences. Importantly, key clinical indicators, including erythrocyte sedimentation rate (14.61 ± 16.87 mm/h vs. 30.69 ± 28.97 mm/h, *P* < 0.001) and glycated hemoglobin (5.48 ± 0.46% vs. 6.05 ± 0.87%, *P* < 0.001), remained significantly higher in the case group after imputation. Differences in arterial blood gas parameters, such as PaO_2_ (81.87 ± 12.07 mmHg vs. 83.92 ± 17.00 mmHg, *P* = 0.212) and PaCO_2_ (40.40 ± 3.99 mmHg vs. 41.30 ± 5.26 mmHg, *P* = 0.117), were attenuated and remained statistically non-significant following data supplementation.

Overall, substantial differences in demographic and clinical features were observed between pneumoconiosis patients with and without TB. Patients in the case group were significantly younger and had lower body mass indices compared with controls. The mean age in the case group was 59.39 ± 10.41 years, compared with 66.01 ± 10.83 years in the control group (*P* < 0.001), accompanied by a markedly lower body mass index (BMI, 24.34 ± 3.53 vs. 21.83 ± 3.24, *P* < 0.001). The proportion of male patients was high in both groups (94.50% in the case group and 95.33% in the control group), with no statistically significant difference (*P* > 0.05). In addition, the case group had significantly fewer years since smoking cessation (6.14 ± 10.00 years vs. 2.19 ± 5.87 years, *P* < 0.001) and a substantially higher proportion of patients without antibiotic use within the preceding 6 months (74.77% vs. 95.41%, *P* < 0.001).

In terms of occupational exposure history, patients in the case group had a significantly shorter duration of dust exposure cessation compared with those in the control group (22.26 ± 12.07 years vs. 13.68 ± 10.70 years, *P* < 0.001), whereas no significant difference was observed in overall exposure duration between the two groups (17.05 ± 8.85 years vs. 15.82 ± 9.76 years, *P* > 0.05). Etiological analysis demonstrated that silicosis was the predominant pneumoconiosis subtype in the case group, accounting for 82.11% of patients, which was markedly higher than in the control group (31.78%, *P* < 0.001). In contrast, coal workers’ pneumoconiosis was more prevalent in the control group (64.49% vs. 15.14%). With respect to disease severity, the proportion of patients with stage III pneumoconiosis was significantly higher in the case group than in the control group (19.63% vs. 40.83%, *P* < 0.001).

Regarding clinical laboratory indicators, patients in the case group exhibited significantly lower hemoglobin levels (144.18 ± 15.79 g/L vs. 129.43 ± 21.36 g/L, *P* < 0.001) and serum albumin concentrations (41.60 ± 3.98 g/L vs. 36.16 ± 5.10 g/L, *P* < 0.001) compared with controls. Conversely, platelet counts (218.63 ± 65.85 × 10^9^/L vs. 250.01 ± 93.75 × 10^9^/L, *P* < 0.001) and inflammatory markers, including C-reactive protein (5.24 ± 11.00 mg/L vs. 31.53 ± 40.50 mg/L, *P* < 0.001) and erythrocyte sedimentation rate (15.66 ± 24.98 mm/h vs. 30.69 ± 28.97 mm/h, *P* < 0.001), were significantly elevated in the case group.

Radiological assessment revealed that the incidence of bilateral lung lesions (61.68% vs. 98.17%, *P* < 0.001), nodules (71.96% vs. 88.53%, *P* < 0.001), cavitary lesions (1.87% vs. 34.40%, *P* < 0.001), and pleural effusion (6.54% vs. 37.61%, *P* < 0.001) was significantly higher among patients with TB than among those without TB. In contrast, no significant differences were observed in the prevalence of pneumothorax (2.80% vs. 1.38%, *P* = 0.646) or bronchiectasis (3.74% vs. 4.13%, *P* > 0.05) between the two groups.

Pathogen analysis indicated that bacterial co-infections in the case group were predominantly caused by bacteria other than *Pseudomonas aeruginosa*, *Staphylococcus aureus*, and *Klebsiella pneumoniae*, with a significantly higher prevalence compared with the control group (14.02% vs. 32.11%, *P* < 0.001). No statistically significant differences were detected between the two groups with respect to fungal infections (0.93% vs. 3.67%, *P* > 0.05) or viral infections (0% vs. 0.92%, *P* > 0.05).

### 3.2 Data set splitting and basic feature description

For model development, the dataset was randomly divided into training and validation sets using R software, with a ratio of 7:3, yielding 227 subjects in the training set and 98 subjects in the validation set. Random seed generation ensured reproducibility of the data partitioning process. Baseline characteristics were well balanced between the two datasets, with no significant differences observed across all variables (*P* > 0.05; Table 4). VIF values for all diagnostic factors were below 5, indicating the absence of significant multicollinearity and confirming their suitability for subsequent modeling (Table 5). Feature selection and LASSO regression modeling were conducted exclusively in the training dataset, while the validation dataset retained the original data distribution to ensure objective assessment of model generalizability and diagnostic performance.

**TABLE 4:** Comparison of baseline characteristics between validation set and training set.

Variable	Total (*n* = 325)	Training set (*n* = 227)	Validation set (*n* = 98)	*P*
Case group	218	154	64	
Control group	107	73	34	
Sex, n (%)				1.0000
Man	308 (94.8)	215 (94.7)	93 (94.9)	
Woman	17 (5.2)	12 (5.3)	5 (5.1)	
Type of disease, n (%)				0.6812
Pneumoconiosis in welders	3 (0.9)	2 (0.9)	1 (1.0)	
Talc pneumoconiosis	1 (0.3)	1 (0.4)	0 (0.0)	
Coal-worker’s pnuemoconiosis	102 (31.4)	70 (30.8)	32 (32.7)	
Asbestosis	4 (1.2)	4 (1.8)	0 (0.0)	
Cement pneumoconiosis	2 (0.6)	2 (0.9)	0 (0.0)	
Silicosis	213 (65.5)	148 (65.2)	65 (66.3)	
Pneumoconiosis stage, n (%)				0.4578
1	132 (40.6)	88 (38.8)	44 (44.9)	
2	83 (25.5)	62 (27.3)	21 (21.4)	
3	110 (33.8)	77 (33.9)	33 (33.7)	
Other underlying lung disease, n (%)				0.4800
COPD	31 (9.5)	22 (9.7)	9 (9.2)	
Carcinoma of the lungs	5 (1.5)	5 (2.2)	0 (0.0)	
Pulmonary interstitial fibrosis	1 (0.3)	1 (0.4)	0 (0.0)	
Rheumatoid secondary fibrosis	1 (0.3)	0 (0.0)	1 (1.0)	
Bronchiectasia	1 (0.3)	1 (0.4)	0 (0.0)	
Bronchial asthma	2 (0.6)	2 (0.9)	0 (0.0)	
Other	19 (5.8)	14 (6.2)	5 (5.1)	
Not have	265 (81.5)	182 (80.2)	83 (84.7)	
Use of antibiotics, n (%)				0.4084
Not used	288 (88.6)	205 (90.3)	83 (84.7)	
≤ 3 Days/month	10 (3.1)	5 (2.2)	5 (5.1)	
≥ 7 Days/month	2 (0.6)	1 (0.4)	1 (1.0)	
3–7 Days/month	25 (7.7)	16 (7.0)	9 (9.2)	
Inhalation of hormones, n (%)				1.0000
≥ 14 Days/month	7 (2.2)	5 (2.2)	2 (2.0)	
Not used	318 (97.8)	222 (97.8)	96 (98.0)	
Germ, n (%)				0.7068
Klebsiella pneumoniae	29 (8.9)	19 (8.4)	10 (10.2)	
Staphylococcus aureus	2 (0.6)	2 (0.9)	0 (0.0)	
Pseudomonas aeruginosa	4 (1.2)	2 (0.9)	2 (2.0)	
Other bacteria	85 (26.2)	58 (25.6)	27 (27.6)	
Not have	205 (63.1)	146 (64.3)	59 (60.2)	
Fungus, n (%)				0.9541
Monilia	9 (2.8)	6 (2.6)	3 (3.1)	
Aspergillus	5 (1.5)	4 (1.8)	1 (1.0)	
Other fungi	9 (2.8)	6 (2.6)	3 (3.1)	
Not have	302 (92.9)	211 (93.0)	91 (92.9)	
Virus, n (%)				1.0000
Other viruses	2 (0.6)	1 (0.4)	1 (1.0)	
Not have	323 (99.4)	226 (99.6)	97 (99.0)	
Location of chest CT lesions, n (%)				0.3879
Bilateral lung	280 (86.2)	192 (84.6)	88 (89.8)	
Right lung	23 (7.1)	17 (7.5)	6 (6.1)	
Left lung	22 (6.8)	18 (7.9)	4 (4.1)	
Tubercle, n (%)				0.3473
Not have	55 (16.9)	35 (15.4)	20 (20.4)	
Have	270 (83.1)	192 (84.6)	78 (79.6)	
Consolidation, n (%)				0.6857
Not have	266 (81.8)	184 (81.1)	82 (83.7)	
Have	59 (18.2)	43 (18.9)	16 (16.3)	
Cavity, n (%)				0.7156
Not have	248 (76.3)	175 (77.1)	73 (74.5)	
Have	77 (23.7)	52 (22.9)	25 (25.5)	
Patching, n (%)				0.9809
Not have	128 (39.4)	90 (39.6)	38 (38.8)	
Have	197 (60.6)	137 (60.4)	60 (61.2)	
Calcification, n (%)				0.3128
Not have	217 (66.8)	156 (68.7)	61 (62.2)	
Have	108 (33.2)	71 (31.3)	37 (37.8)	
Pleural effusion, n (%)				0.9272
Not have	236 (72.6)	164 (72.2)	72 (73.5)	
Have	89 (27.4)	63 (27.8)	26 (26.5)	
Aerothorax, n (%)				0.7813
Not have	319 (98.2)	222 (97.8)	97 (99.0)	
Have	6 (1.8)	5 (2.2)	1 (1.0)	
Bronchiectasis, n (%)				0.7205
Not have	312 (96.0)	219 (96.5)	93 (94.9)	
Have	13 (4.0)	8 (3.5)	5 (5.1)	
Age, mean ± SD	61.57 ± 10.99	62.18 ± 11.07	60.15 ± 10.70	0.1270
BMI, mean ± SD	22.65 ± 3.54	22.42 ± 3.53	23.19 ± 3.51	0.0704
Years of dust exposure, mean ± SD	16.22 ± 9.47	16.22 ± 9.52	16.22 ± 9.39	0.9999
Years of dust exposure cessation, mean ± SD	16.51 ± 11.86	17.00 ± 12.25	15.36 ± 10.87	0.2510
Smoking index, mean ± SD	295.65 ± 358.02	296.53 ± 349.46	293.62 ± 378.95	0.9466
Years of smoking cessation, mean ± SD	3.49 ± 7.69	3.47 ± 7.29	3.52 ± 8.58	0.9630
White blood cells, mean ± SD	6.79 ± 2.40	6.72 ± 2.50	6.96 ± 2.16	0.4081
Hemoglobin, mean ± SD	134.29 ± 20.87	133.94 ± 21.27	135.09 ± 20.00	0.6487
Platelets, mean ± SD	239.68 ± 86.74	243.24 ± 89.62	231.42 ± 79.50	0.2600
NE#, mean ± SD	6.81 ± 11.29	7.10 ± 11.80	6.15 ± 10.03	0.4860
LY#, mean ± SD	2.16 ± 4.15	2.27 ± 4.70	1.92 ± 2.47	0.4877
MO#, mean ± SD	0.75 ± 1.71	0.80 ± 1.93	0.65 ± 1.02	0.4685
CRP, mean ± SD	22.87 ± 35.94	23.00 ± 36.41	22.57 ± 34.99	0.9211
PCT, mean ± SD	0.11 ± 0.26	0.12 ± 0.30	0.10 ± 0.11	0.4158
ESR, mean ± SD	25.40 ± 26.69	25.24 ± 26.45	25.77 ± 27.39	0.8680
ALT, mean ± SD	21.53 ± 25.71	22.16 ± 29.04	20.09 ± 15.47	0.5068
AST, mean ± SD	25.41 ± 21.76	26.00 ± 24.20	24.03 ± 14.67	0.4565
Albumin, mean ± SD	37.95 ± 5.40	37.70 ± 5.36	38.55 ± 5.46	0.1912
Creatinine, mean ± SD	68.64 ± 15.95	68.06 ± 16.07	69.99 ± 15.66	0.3192
Urea nitrogen, mean ± SD	5.22 ± 1.88	5.20 ± 1.93	5.29 ± 1.74	0.6888
PH, mean ± SD	7.41 ± 0.17	7.41 ± 0.20	7.41 ± 0.03	0.7994
PaO_2_, mean ± SD	83.24 ± 15.56	83.84 ± 15.62	81.88 ± 15.41	0.2979
PaCO2, mean ± SD	41.00 ± 4.89	40.90 ± 4.93	41.24 ± 4.83	0.5753
PaO_2_/FiO_2_, mean ± SD	358.36 ± 59.32	360.18 ± 60.87	354.15 ± 55.64	0.4011
Lac, mean ± SD	1.49 ± 1.29	1.55 ± 1.51	1.34 ± 0.42	0.1736
HbA1c, mean ± SD	5.86 ± 0.81	5.87 ± 0.77	5.85 ± 0.88	0.8545

M ± SD: M, mean; SD, standard deviation.

**TABLE 5:** Collinearity diagnosis table.

Variable name	VIF
Age	2.974
Sex	1.321
BMI	1.459
Type of etiology	2.899
Exposure duration	1.757
Stage of pneumoconiosis	1.411
Duration of dust exposure cessation	2.519
Other underlying lung diseases	3.610
Smoking index	1.170
Years of abstinence	1.157
Use of antibiotics	1.844
Inhalation of hormones	1.552
White blood cell	1.639
Hemoglobin	2.193
Blood cells	1.412
NE#	2.414
LY#	2.100
MO#	1.915
CRP	2.727
PCT	1.422
ESR	2.041
ALT	1.925
AST	1.913
Albumin	2.566
Creatinine	1.588
Usea nitrogen	1.472
PH	1.144
PaO_2_	1.678
PaCO2	1.181
PaO_2_/FiO_2_	2.169
Lac	1.070
Glycosylated hemoglobin	1.228
Germ	2.074
Fungus	1.612
Virus	1.064
Lesion location	1.778
Tubercle	1.275
Consolidation	1.140
Cavity	1.255
Patching	1.174
Calcification	1.170
Pleural effussion	1.276
Aerothorax	1.098
Bronchiectasia	1.337

### 3.3 Selection of variables for the diagnostic model

In the univariable analysis of the training dataset, 31 candidate diagnostic factors with *P* < 0.10 were identified and subsequently entered into LASSO regression for further dimensionality reduction and variable selection. LASSO regression applies a penalty to the absolute magnitude of regression coefficients, thereby shrinking less informative coefficients toward zero and retaining only the most relevant diagnostic factors.

**FIGURE 1:**
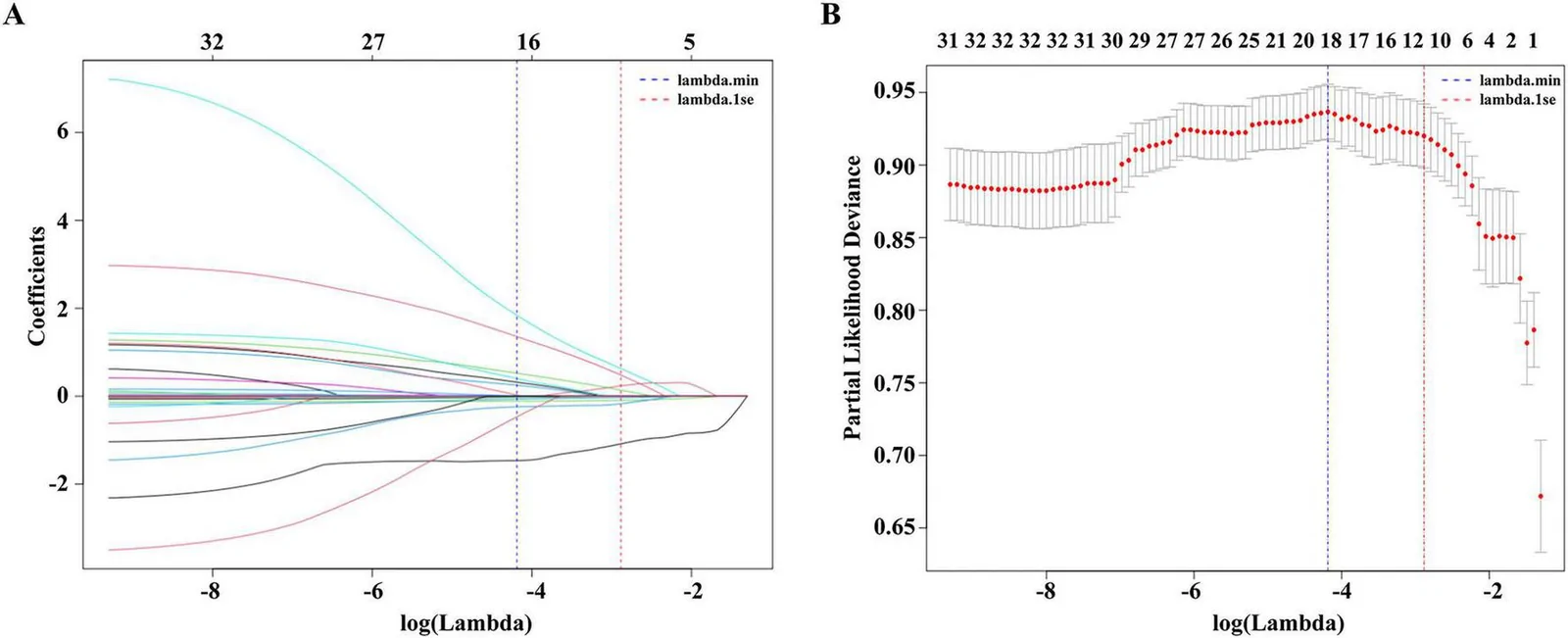
Lasso fitting diagram. **(A)** Trajectory of Lasso fitting coefficients, with the logarithm of the regularization parameter (log λ) on the horizontal axis and the standardized regression coefficients on the vertical axis; **(B)**. MSE plot of Lasso regression fitting. The *x*-axis represents log λ, allowing visualization of model behavior across multiple orders of magnitude, while the *y*-axis depicts the cross-validated MSE. The red curve indicates the trend of diagnostic error as λ varies, and the gray shaded area denotes the standard error of the MSE. MSE, mean squared error.

Figure 1A illustrates the coefficient path plot. As λ increases, the regularization strength intensifies, progressively shrinking more coefficients toward zero and enabling variable selection. Each trajectory represents the coefficient path of an individual variable across different values of λ, and the coefficients of retained variables gradually diminish as λ increases.

Figure 1B presents the LASSO cross-validation curve. Two optimal λ values are highlighted: λ_*min*_, which corresponds to the minimum mean squared error (MSE) and retained 19 variables, and λ_1s*e*_, which identifies a more parsimonious model within one standard error of the minimum MSE and retained 10 variables. To balance diagnostic performance and model complexity, variables corresponding to λ_1s*e*_ were selected for subsequent modeling. Using this criterion, LASSO regression identified the following 10 variables: etiological type, serum albumin, PaO_2_/FiO_2_ ratio, C-reactive protein, glycated hemoglobin, calcification, bilateral lung lesions, cavitation, neutrophil count, and lymphocyte count.

To further reduce the potential instability associated with single LASSO selection, nested cross-validation was employed to assess the robustness of feature selection. Across five outer cross-validation folds, 10 variables, including etiological type, prior antibiotic use, bilateral lung lesions, cavitation, calcification, pleural effusion, duration of dust exposure cessation, serum albumin, PaO_2_/FiO_2_ ratio, and glycated hemoglobin, were selected in all validation rounds, with selection frequencies exceeding 80%, indicating high stability. The mean AUC obtained from nested cross-validation was 0.928, with a standard deviation of 0.025, demonstrating excellent discriminative performance and generalizability of the feature set.

Based on the combined results of LASSO regression and nested cross-validation, a final set of 10 variables was selected for construction of the diagnostic model: etiological type, serum albumin, PaO_2_/FiO_2_ ratio, glycated hemoglobin, calcification, bilateral lung lesions, cavitation, prior antibiotic use, pleural effusion, and duration of dust exposure cessation.

### 3.4 Machine learning results

The hyperparameters for each machine learning algorithm were optimized using grid search, with the final parameter settings summarized in Table 6.

**TABLE 6:** Parameters settings of different machine learning models.

Model category	Parameter configuration
XGBoost	nrounds = 62; max_depth = 6; eta = 0.1
RF	ntree = 500; mtry = 3
Tree	cp = 0.003
LightGBM	num_leaves = 7; max_depth = 5; learning_rate = 0.05; min_data_in_leaf = 20; lambda_l1 = 0; lambda_l2 = 0; scale_pos_weight = 73/154
KNN	K = 11
SVM	Sigma = 0.03125; C = 4
GBM	Interaction.depth = 5; n.trees = 200; shrinkage = 0.05; n.minobsinnode = 20
LR	Do not participate in parameter tuning

#### 3.4.1 Performance of training set models

The ten stable variables identified through nested cross-validation were used to construct diagnostic models using eight machine learning algorithms: logistic regression (LR), RF, decision tree (Tree), XGBoost, k-nearest neighbors (KNN), gradient boosting machine (GBM), LightGBM, and support vector machine (SVM). Model performance was evaluated on both the training and validation datasets, and receiver operating characteristic (ROC) curves were generated for each model. Detailed performance metrics for all models are presented in Tables 7, 8.

**TABLE 7:** Features of the prediction model for each machine learning training set.

Model	AUC (95%CI)	Accuracy	Sensitivity	Specificity	F1	PPV	NPV	Brier score	NRI	Delong test *P*
LR	0.946 (0.910, 0.981)	0.872	0.883	0.849	0.904	0.925	0.775	0.075	−0.260 (−0.372, −0.132)	0.003
RF	0.997 (0.994, 1.000)	0.978	0.981	0.973	0.984	0.987	0.959	0.043	Reference	Reference
Tree	0.903 (0.853, 0.905)	0.899	0.955	0.781	0.927	0.902	0.891	0.079	−0.141 (−0.271, −0.018)	<0.001
XGBoost	0.941 (0.905, 0.977)	0.899	0.942	0.808	0.927	0.912	0.868	0.083	−0.308 (−0.428, −0.197)	0.002
KNN	0.926 (0.889, 0.963)	0.872	0.909	0.795	0.906	0.903	0.806	0.086	−0.289 (−0.408, −0.172)	<0.001
GBM	0.984 (0.971, 0.996)	0.934	1.000	0.795	0.954	0.911	1.000	0.047	−0.064 (−0.154, 0.026)	0.009
LightGBM	0.996 (0.992, 1.000)	0.952	0.929	1.000	0.963	1.000	0.869	0.034	0.318 (−0.043, 0.115)	0.337
SVM	0.965 (0.941, 0.983)	0.894	0.877	0.932	0.918	0.964	0.782	0.074	−0.223 (−0.335, −0.111)	<0.001

AUC, area under the curve; PPV, positive predictive value; NPV, negative predictive value.

**TABLE 8:** Features of the machine learning prediction model on each validation set.

Model	AUC (95%CI)	Accuracy	Sensitivity	Specificity	F1	PPV	NPV	Brier score	NRI	Delong test *P*
LR	0.909 (0.852, 0.967)	0.847	0.875	0.794	0.882	0.889	0.771	0.118	−0.022 (−0.236, 0.188)	0.850
RF	0.905 (0.833, 0.977)	0.898	0.938	0.824	0.923	0.909	0.875	0.109	Reference	Reference
Tree	0.833 (0.745, 0.836)	0.867	0.922	0.765	0.901	0.881	0.839	0.136	0.129 (−0.069, 0.334)	0.028
XGBoost	0.899 (0.838, 0.960)	0.827	0.891	0.706	0.870	0.851	0.774	0.124	−0.081 (−0.274, 0.133)	0.972
KNN	0.866 (0.795, 0.937)	0.755	0.813	0.647	0.813	0.813	0.647	0.131	−0.155 (−0.349, 0.055)	0.238
GBM	0.883 (0.804, 0.961)	0.857	0.969	0.647	0.899	0.838	0.917	0.113	0.143 (−0.011, 0.287)	0.116
LightGBM	0.909 (0.844, 0.973)	0.837	0.828	0.853	0.869	0.914	0.725	0.106	0.076 (−0.043, 0.198)	0.801
SVM	0.886 (0.806, 0.952)	0.796	0.781	0.824	0.833	0.893	0.667	0.132	−0.079 (−0.267, 0.102)	0.311

AUC, area under the curve; PPV, positive predictive value; NPV, negative predictive value.

In the training set, the RF model demonstrated superior performance, achieving an AUC of 0.997 (95% CI: 0.994–1.000). Compared with RF, all other models had significantly lower AUCs according to Delong’s test (*P* < 0.05), indicating RF’s statistical superiority. Using RF as the reference, the training set AUCs for the remaining models were: LightGBM 0.996 (95% CI: 0.992–1.000), GBM 0.984 (95% CI: 0.971–0.996), SVM 0.965 (95% CI: 0.941–0.983), LR 0.946 (95% CI: 0.910–0.981), XGBoost 0.941 (95% CI: 0.905–0.977), KNN 0.926 (95% CI: 0.889–0.963), and Tree 0.903 (95% CI: 0.853–0.905). KNN exhibited the poorest training set performance.

#### 3.4.2 ROC curves in training and validation sets

**FIGURE 2:**
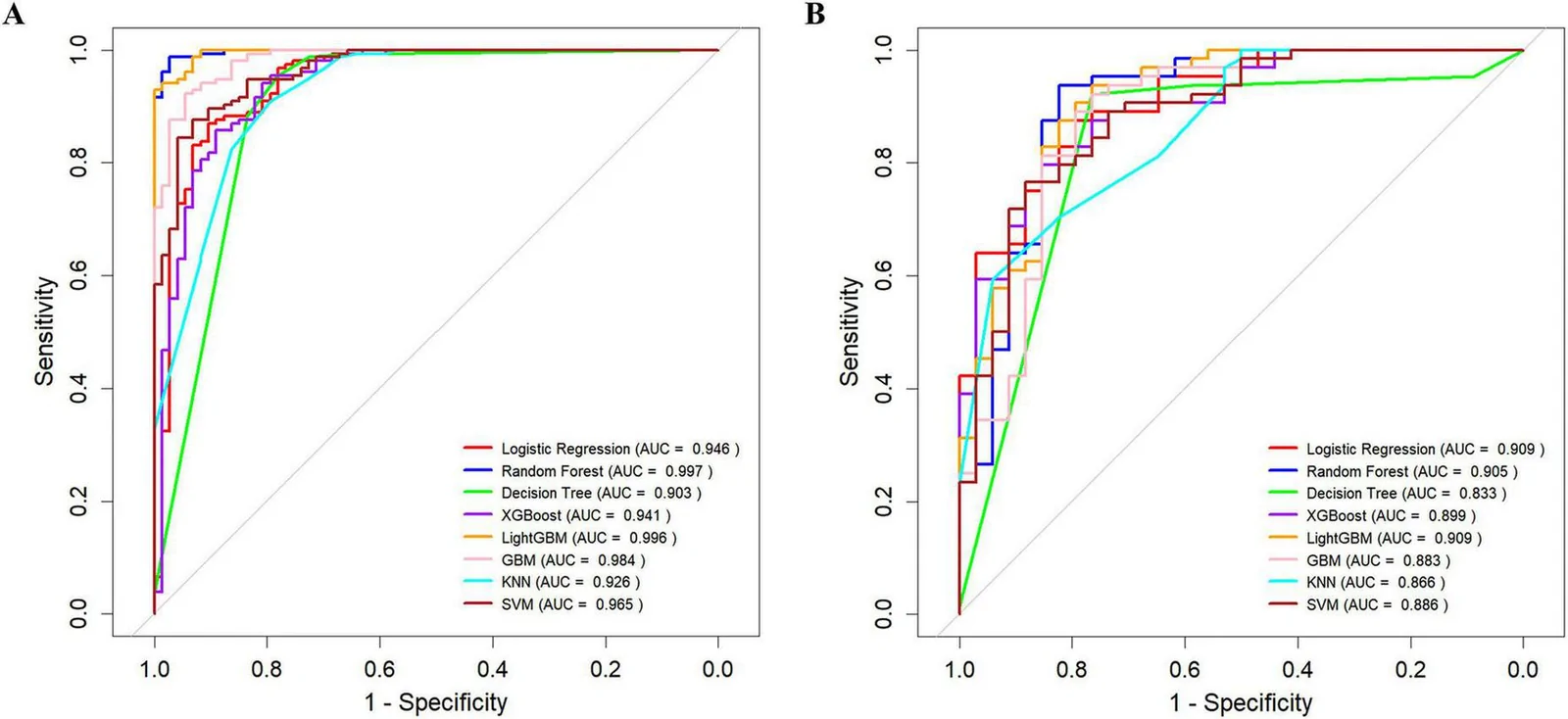
ROC curves for different machine learning models on the training and validation sets. **(A)** Training set. **(B)** Validation set. ROC, receiver operating characteristic.

ROC analysis confirmed RF as the best-performing model in both the training and validation datasets (Figures 2). In the training set, RF achieved the highest AUC (0.997, 95% CI: 0.994–1.000), followed by LightGBM (0.996, 95% CI: 0.992–1.000) and GBM (0.984, 95% CI: 0.971–0.996).

In the validation set, RF maintained strong diagnostic performance with an AUC of 0.905 (95% CI: 0.833–0.977), comparable to LR (0.909, 95% CI: 0.852–0.967) and LightGBM (0.909, 95% CI: 0.844–0.973). Delong’s test revealed no statistically significant differences between RF and LR, LightGBM, XGBoost, GBM, or SVM (All *P* > 0.05), indicating comparable discriminative ability among these models. The remaining models exhibited lower performance, with XGBoost (0.899, 95% CI: 0.838–0.960), GBM (0.883, 95% CI: 0.804–0.961), SVM (0.886, 95% CI: 0.806–0.952), KNN (0.866, 95% CI: 0.795–0.937), and Tree (0.833, 95% CI: 0.745–0.836) ranking lower.

Although LR and LightGBM showed slightly higher validation AUCs than RF (0.909 vs. 0.905), RF demonstrated a more balanced overall performance, with superior sensitivity (0.981 in training vs. 0.938 in validation) and good specificity (0.973 in training vs. 0.824 in validation) across both datasets. LightGBM, while showing acceptable sensitivity (0.828) and specificity (0.853) on the validation set, had noticeably lower sensitivity than RF. LR also exhibited relatively low specificity (0.794) and negative diagnostic value (0.771). Notably, the RF model’s AUC decreased by only 0.092 from training to validation, a smaller reduction than most other models, indicating strong generalization and minimal overfitting.

**FIGURE 3:**
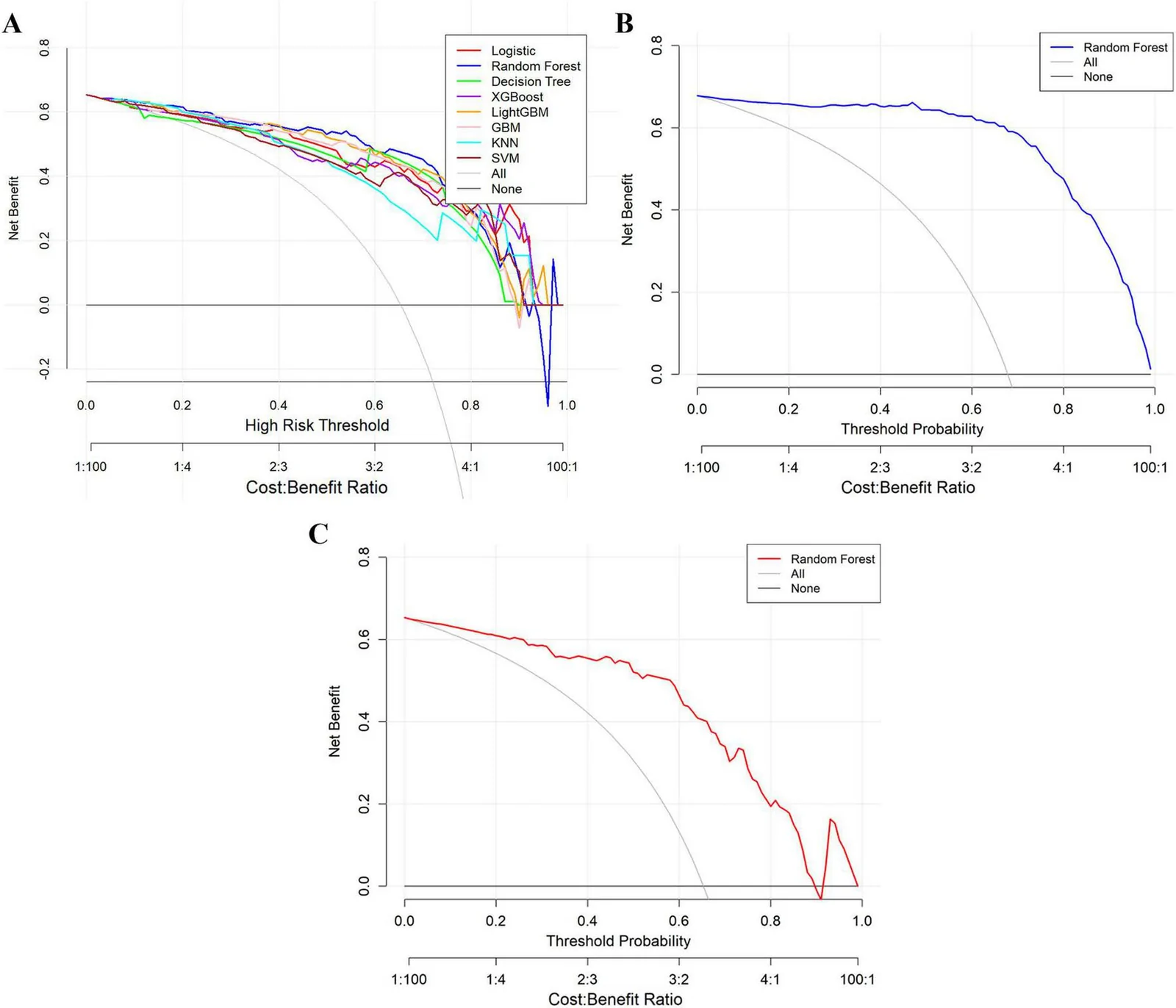
DCA curves for different machine learning models. **(A)** DCA curves of various machine learning diagnostic models. In the DCA plots, the lines for each model indicate the net benefit across varying probability thresholds. The “All” strategy (gray line) represents a hypothetical approach in which every patient receives intervention regardless of model diagnostic, while the “None” strategy (black line) represents no intervention for any patient. **(B)** DCA curve of the RF diagnostic model training set. **(C)** DCA curve of RF diagnostic model validation set. DCA, decision curve analysis; RF, random forest.

RF also achieved high F1 scores (0.984 in training vs. 0.923 in validation) and low Brier scores (0.043 vs. 0.109), reflecting good calibration. Decision curve analysis further confirmed that RF offered the highest net clinical benefit across relevant threshold probabilities (Figure 3A). Considering AUC stability, sensitivity-specificity balance, generalization ability, calibration, and clinical applicability, the RF model was identified as the optimal diagnostic model for TB risk in pneumoconiosis patients.

#### 3.4.3 Decision curve analysis and optimal model evaluation

##### 3.4.3.1 Decision curve analysis of machine learning models

DCA was performed to assess the clinical utility of the different machine learning models. In the DCA plot, the horizontal axis represents the probability threshold at which the model diagnoses a positive outcome (i.e., the likelihood of TB), guiding whether clinical action (e.g., intervention or treatment) should be taken. The vertical axis represents the net benefit, quantifying the additional advantage of using the model for decision-making compared with strategies that do not incorporate model diagnostic.

As shown in Figure 3A, the RF model consistently provides a higher net benefit than both the “All” and “None” strategies across most threshold ranges. This demonstrates that applying the RF model in clinical decision-making can achieve superior risk stratification and more effective intervention planning compared with other machine learning models or uniform treatment strategies.

##### 3.4.3.2 Evaluation of the optimal model

**FIGURE 4:**
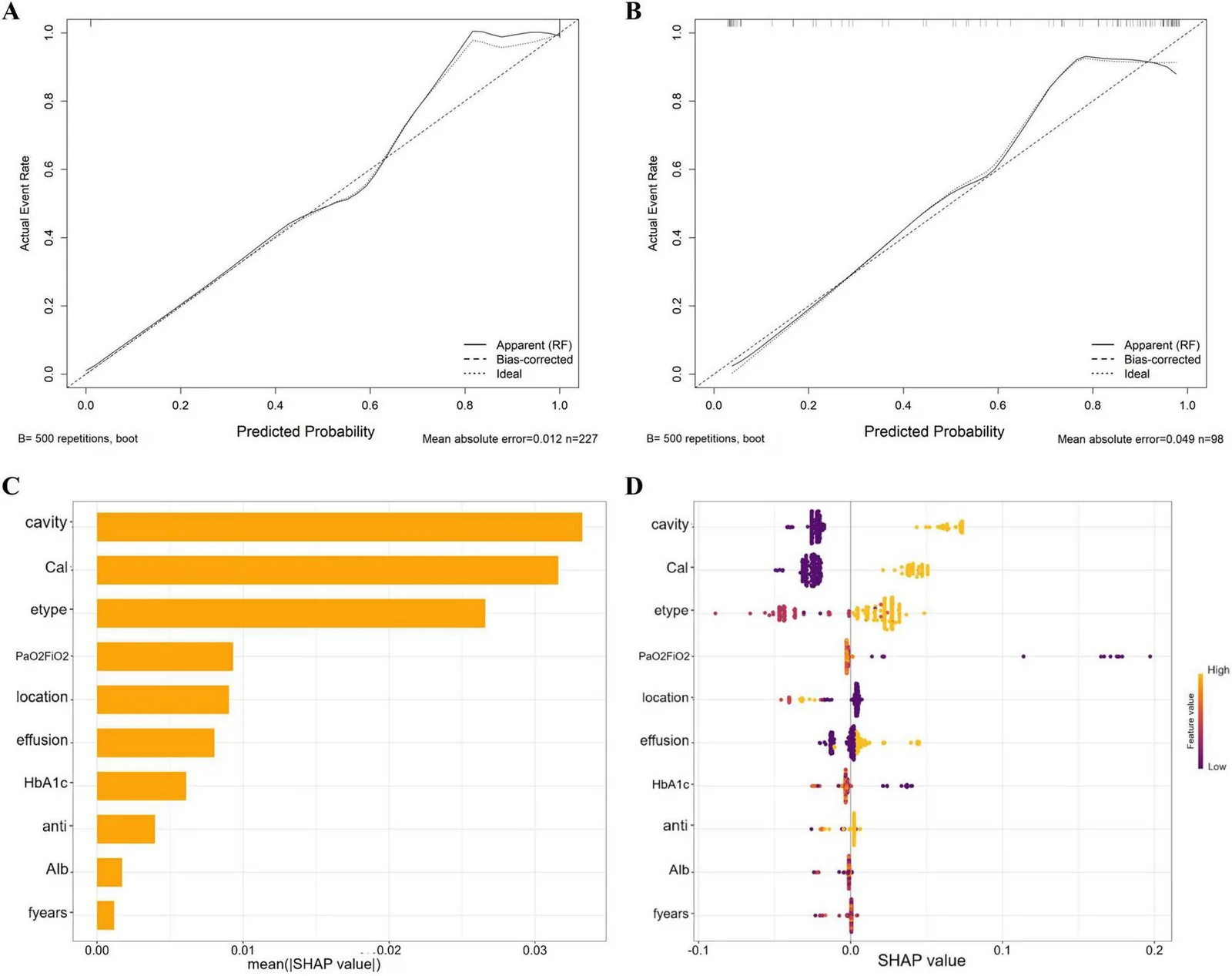
RF diagnostic model evaluation. **(A)** Calibration curve of RF diagnostic model training set; **(B)** Calibration curve of RF diagnostic model validation set. On these plots, the horizontal axis represents the diagnosed probability of TB, while the vertical axis indicates the actual observed probability. The red dashed line represents perfect calibration, where diagnosed and observed probabilities are identical; **(C)** Bar chart of RF model feature importance. The vertical axis lists the features ranked by importance, while the horizontal axis represents the mean absolute SHAP values. Higher positions on the vertical axis indicate greater feature significance; **(D)** Bee colony diagram of RF model feature importance, where each point represents a patient and colors reflect high or low SHAP values. RF, random forest; TB, tuberculosis; SHAP, shapley additive explanations.

Figures 3B,C illustrate the DCA curves for the RF model in the training and validation sets, respectively. In both datasets, the RF model maintains higher net benefit than the control strategies across nearly the entire range of clinically relevant thresholds, confirming its robust clinical applicability. Calibration curves further assessed the agreement between diagnosed probabilities and observed outcomes for the RF model (Figures 4A,B). In the training set, the RF model’s diagnosed probabilities closely matched the actual outcomes, with observed probabilities remaining below 0.6. In the validation set, the model demonstrated even better alignment, maintaining strong agreement after 500 bootstrap resamples. The mean absolute error (MAE) was 0.012 for the training set and 0.049 for the validation set, indicating minimal deviation between diagnosed and observed probabilities. These results confirm that the RF model not only provides high discriminative ability but also exhibits excellent calibration, reinforcing its reliability for clinical application. Overall, the RF model demonstrates superior performance in terms of both clinical decision-making utility and diagnostic accuracy, making it the optimal choice for diagnosing TB risk in pneumoconiosis patients.

##### 3.4.3.3 Explainable analysis of the optimal model using SHAP

The SHAP method was applied to interpret the RF model and quantify the contribution of each feature to the diagnostic of TB in pneumoconiosis patients. In the summary plot (Figure 4C), cavitation emerged as the most influential diagnostic factor, playing a central role in determining the likelihood of PTB. Other highly impactful features included calcification, etiology type, PaO_2_/FiO_2_, and lesion location (bilateral lung lesions).

Figure 4D presents a feature-level SHAP visualization for individual samples. Samples with higher SHAP values correspond to greater diagnosed risk of TB. This analysis confirms that cavitation, calcification, etiology type, PaO_2_/FiO_2_, and lesion location (bilateral lung lesions) are strongly associated with the model’s output, with cavitation identified as the key diagnostic factor.

**FIGURE 5:**
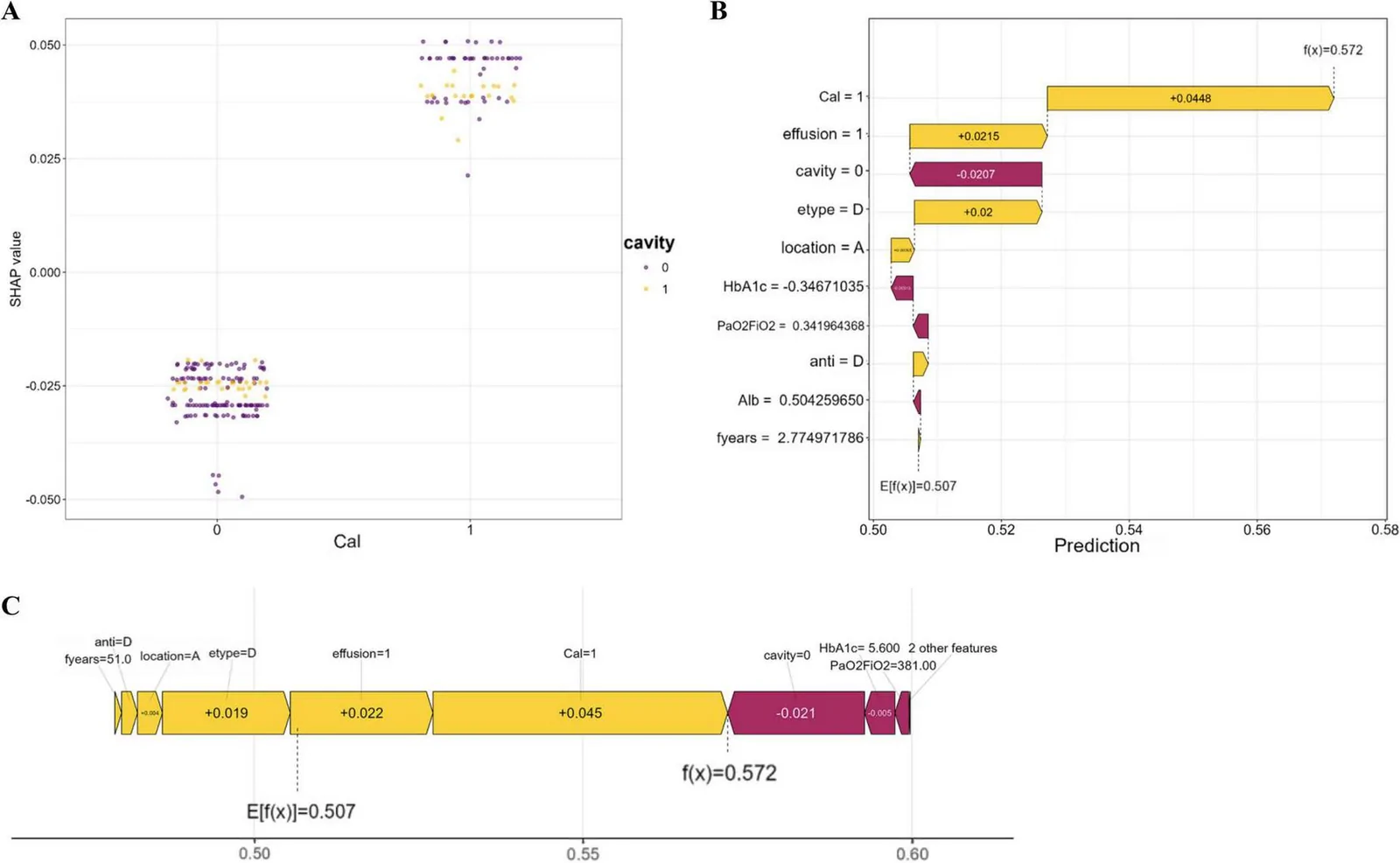
Individualized diagnostic results of the RF model based on SHAP. **(A)** Partial correlation dependence graph of calcification and cavity. Each point represents a sample, with color coding indicating CRP levels. **(B)** Single-sample waterfall diagram of the RF model. **(C)** RF single sample feature map. Each rectangle represents the contribution of a single feature to the model’s diagnostic. The horizontal axis shows SHAP values, with left-pointing (purple) rectangles indicating features that decrease diagnosed risk and right-pointing (yellow) rectangles indicating features that increase diagnosed risk. RF, random forest; CRP, C-reactive protein; SHAP, shapley additive explanations.

Partial dependence analysis (Figure 5A) further explored the interaction between cavitation, calcification, and SHAP values. The results demonstrate that higher SHAP values for cavitation and calcification positively contribute to the diagnosed risk, confirming their synergistic effect in influencing TB susceptibility.

Figures 5B,C illustrate individualized diagnostic from the RF model using SHAP. The baseline value, E[f(x)], represents the mean diagnosed probability (0.507) across all samples. In Figure 5B, six features exert a positive influence while four features contribute negatively. The cumulative effect of these contributions increases the diagnosed probability from the baseline of 0.507 to a final value of 0.572, demonstrating how the model integrates multiple feature contributions to generate individualized risk diagnostic.

## 4 Discussion

The development of PTB in patients with pneumoconiosis is influenced by both occupational and non-occupational factors (9, 12–14). Inhaled silica particles trigger alveolar inflammatory responses and impair alveolar macrophage function. Persistent inflammation promotes fibroblast proliferation and activation, collagen deposition, and the formation of silicotic nodules and pulmonary fibrosis. These pathological changes hinder the clearance of *M. tuberculosis*, thereby facilitating its colonization, growth, and replication. In this study, patients with pneumoconiosis complicated by TB were younger at onset (66.01 ± 10.83 years vs. 59.39 ± 10.41 years, *P* < 0.001) and exhibited poorer nutritional status, as indicated by lower BMI (24.34 ± 3.53 vs. 21.83 ± 3.24), hemoglobin (144.18 ± 15.79 g/L vs. 129.43 ± 21.36 g/L), and albumin levels (41.60 ± 3.98 g/L vs. 36.16 ± 5.10 g/L; all *P* < 0.001). An earlier onset of pneumoconiosis may result in greater cumulative lung damage from dust exposure, including more extensive silicotic nodules and pulmonary fibrosis, and may be associated with greater suppression of immune function. These patients also had shorter durations of smoking cessation (6.14 ± 10.00 years vs. 2.19 ± 5.87 years, *P* < 0.001). Our findings are consistent with previously published studies.

Regarding occupational exposure, silicosis was more prevalent in the case group (31.78% vs. 82.11%, *P* < 0.001), and a higher proportion had stage III pneumoconiosis (19.63% vs. 40.83%, *P* < 0.001). A meta-analysis of cohort studies indicating that silicosis and occupational silica exposure are associated with an increased risk of TB (20). The duration of dust exposure cessation was shorter in the case group, which may indicate that within the TB group, a subset of individuals possessed a higher biological susceptibility to dust-induced lung injury. These susceptible individuals might progress to severe pneumoconiosis and its complications, such as TB, after a period of high-intensity occupational exposure. This highlights that factors beyond cumulative dose, including host susceptibility and the rate of disease progression, are critical determinants of TB risk in this population.

Using LASSO regression and nested cross-validation, ten key diagnostic factors of TB in pneumoconiosis patients were identified: type of pneumoconiosis etiology, albumin, oxygenation index (PaO_2_/FiO_2_), glycated hemoglobin (HbA1c), calcification, bilateral lung lesions, cavitation, prior use of antibiotics, pleural effusion, and duration of dust exposure cessation. Occupational factors, including etiology type and duration of dust exposure cessation, alongside nutritional and metabolic indicators (albumin and HbA1c), collectively influence TB risk. The PaO_2_/FiO_2_ ratio reflects the severity of lung function impairment in pneumoconiosis; a lower oxygenation index indicates more severe pulmonary dysfunction. HbA1c reflects long-term glycemic control, and patients with poor glycemic control may exhibit impaired immune function. Prior antibiotic use can indirectly reflect the immune status of patients. Radiological features, such as cavitation, lesion location (bilateral lung lesions), calcification, and pleural effusion, reflect disease severity and play a critical role in risk stratification.

Multiple machine learning algorithms, including LR, KNN, Decision Tree, RF, SVM, XGBoost, LightGBM, and GBM, were constructed to diagnose TB occurrence. All models demonstrated strong diagnostic capacity, with the RF model achieving the highest overall performance (training AUC = 0.997, 95% CI: 0.994–1.000; validation AUC = 0.905, 95% CI: 0.833–0.977). Explainable analysis using SHAP highlighted cavity, calcification, etiological type, PaO_2_/FiO_2_, and lesion location (bilateral lung lesions) as the most influential features, providing transparent insights into the model’s decision-making process. It is important to acknowledge the marked baseline differences between the pneumoconiosis patients with and without TB in our cohort, including age, BMI, albumin levels, pneumoconiosis type, and disease stage. These differences suggest that the identified predictors, such as lower serum albumin and a higher proportion of stage III pneumoconiosis, may reflect a profile of greater overall disease severity and poorer general health status, which in turn creates a permissive environment for TB development. Therefore, while our model identifies variables strongly associated with concomitant TB, some may be proxies for a debilitated state rather than TB-specific pathognomonic features. This does not diminish their utility within the predictive model but highlights that the model’s predictions are for TB risk within a context of advanced pneumoconiosis and its comorbidities.

Several limitations should be acknowledged. First, this study employed a retrospective design, which may introduce selection bias. Second, misclassification of TB is possible due to the disease’s complexity and heterogeneous clinical presentations, which may affect the model’s diagnostic performance. Third, although the machine learning model demonstrated strong performance in internal validation, the lack of independent external validation warrants further confirmation of its generalizability and diagnostic stability. Fourth, the final case-control ratio deviated from the initially planned 1:1 design due to the retrospective nature of data availability, which may introduce some selection bias. Finally, the data were collected from a limited geographic region, and the sample size was modest, which may affect the generalizability of the findings. Future research should include multicenter cohorts with larger sample sizes to validate and refine the model, assess its performance across diverse populations, and further enhance diagnostic accuracy.

## 5 Conclusion

For patients with occupational pneumoconiosis, the risk of developing PTB can be effectively diagnosed using a combination of occupational history, laboratory indicators, and radiographic findings. Among the models evaluated, the RF algorithm demonstrated the highest diagnostic accuracy and stability, which is helpful for early auxiliary diagnosis, personalized monitoring, and timely intervention.

## Data Availability

The original contributions presented in the study are included in the article, further inquiries can be directed to the corresponding authors.
