# Correction: Construction and application of a diagnostic model for tuberculosis in patients with pneumoconiosis

**DOI:** 10.3389/fmed.2026.1942373

**Published:** 2026-08-07

**Authors:** Shuang Qu, Shiping Hu, Jun Zhu, Huan Ye

**Affiliations:** 1 Department of Respiratory and Critical Care Medicine, Beijing Chest Hospital, Capital Medical University, Beijing, China; 2 Department of Respiratory and Critical Care Medicine, Fuxing Hospital, Capital Medical University, Beijing, China; 3 Department of Occupational Diseases, Shilong Hospital, National Center for Occupational Safety and Health, National Health Commission, Beijing, China

**Keywords:** machine learning, pneumoconiosis, risk diagnostic model, risk stratification, tuberculosis

In the abstract, the expression “duration of dust exposure” is incorrectly used in the first and second sentences of the Results section. This has been corrected to read: “duration of dust exposure cessation.”

There was a mistake in Tables 1, 2 as published. The term “Dust removal period” in the Variable column is incorrect. This has been corrected to read: “Duration of dust exposure cessation.”

There was a mistake in Table 3 as published. The term “Mean ± SD of dust removal period” in the Variables column is incorrect. This has been corrected to read: “Years of dust exposure cessation, mean ± SD.”

There was a mistake in Table 4 as published. The term “Dust removal period, mean ± SD” in the Variable column is incorrect. This has been corrected to read: “Years of dust exposure cessation, mean ± SD.”

There was a mistake in Table 5 as published. The term “Dust removal period” in the Variable name column is incorrect. This has been corrected to read: “Duration of dust exposure cessation.”

The expression “duration of dust removal duration” is incorrectly used in the Section, **3 Results**, “*3.1 Description of baseline characteristics*,” paragraph 2, the second sentence.

A correction has been made to the Section, **3 Results**, “*3.1 Description of baseline characteristics*,” paragraph 2, the second sentence:

replace “duration of dust removal duration” in the original text with “duration of dust exposure cessation.”

The expression “duration of dust exposure” is incorrectly used in the Section, **3 Results**, “*3.1 Description of baseline characteristics*,” paragraph 5, the first sentence.

A correction has been made to the Section, **3 Results**, “*3.1 Description of baseline characteristics*,” paragraph 5, the first sentence:

replace “ duration of dust exposure ” in the original text with “duration of dust exposure cessation.”

The expression “duration of dust exposure” is incorrectly used in the Section, **3 Results**, “*3.3 Selection of variables for the diagnostic model*,” paragraph 4, the second sentence.

A correction has been made to the Section, **3 Results**, “*3.3 Selection of variables for the diagnostic model*,” paragraph 4, the second sentence:

replace “duration of dust exposure ” in the original text with “duration of dust exposure cessation.”

The expression “duration of dust exposure” is incorrectly used in the Section, **3 Results**, “*3.3 Selection of variables for the diagnostic model*,” paragraph 5.

A correction has been made to the Section, **3 Results**, “*3.3 Selection of variables for the diagnostic model*,” paragraph 5:

replace “duration of dust exposure” in the original text with “duration of dust exposure cessation.”

The expression “the duration of removal duration” is incorrectly used in the Section, **4 Discussion**, paragraph 2.

A correction has been made to the Section, **4 Discussion**, paragraph 2:

“Regarding occupational exposure, silicosis was more prevalent in the case group (31.78% vs. 82.11%, *P* < 0.001), and a higher proportion had stage III pneumoconiosis (19.63% vs. 40.83%, *P* < 0.001). A meta-analysis of cohort studies indicating that silicosis and occupational silica exposure are associated with an increased risk of TB (20). The duration of dust exposure cessation was shorter in the case group, which may indicate that within the TB group, a subset of individuals possessed a higher biological susceptibility to dust-induced lung injury. These susceptible individuals might progress to severe pneumoconiosis and its complications, such as TB, after a period of high-intensity occupational exposure. This highlights that factors beyond cumulative dose, including host susceptibility and the rate of disease progression, are critical determinants of TB risk in this population.”

The expression “duration of dust exposure” is incorrectly used in the Section, **4 Discussion**, paragraph 3, the first sentence.

A correction has been made to the Section, **4 Discussion**, paragraph 3, the first sentence:

replace “duration of dust exposure ” in the original text with “duration of dust exposure cessation.”

The expression “dust exposure duration” is incorrectly used in the Section, **4 Discussion**, paragraph 3, the second sentence.

A correction has been made to the Section, **4 Discussion**, paragraph 3, the second sentence:

replace “dust exposure duration” in the original text with “duration of dust exposure cessation.”

The original version of this article has been updated.

